# The Improvement of Sepsis-Associated Encephalopathy by P2X7R Inhibitor through Inhibiting the Omi/HtrA2 Apoptotic Signaling Pathway

**DOI:** 10.1155/2022/3777351

**Published:** 2022-01-27

**Authors:** Kaifang Wang, Meiyan Sun, Zhaodong Juan, Jianxin Zhang, Yingui Sun, Guizhi Wang, Chunling Wang, Yanjing Li, Wenwen Kong, Lulu Fan, Yue Zhang, Hongxiang Zhao, Xiaoyong Zhao

**Affiliations:** ^1^Shandong Provincial Medicine and Health Key Laboratory of Clinical Anesthesia, School of Anesthesiology, Weifang Medical University, 261053 Weifang, Shandong, China; ^2^Department of Anesthesiology, Suzhou Benkey Hospital, 215009 Suzhou, Jiangsu, China; ^3^The Affiliated Hospital of Weifang Medical University, 261021 Weifang, Shandong, China

## Abstract

The pathogenesis of sepsis-associated encephalopathy (SAE) involves many aspects, including intracellular peroxidative stress damage, mitochondrial dysfunction, and cell apoptosis. In this study, we mainly explored the influence of P2X7R on the cognitive function of SAE and its molecular mechanism. We established a sepsis model using lipopolysaccharide (LPS) stimulation, followed by an assessment of cognitive function using Morris water maze, and then Western Blot was used to analyze the expression of tight junction proteins ZO-1 and Occludin in the hippocampus of mice. TUNEL assay was used to analyze the apoptosis of brain cells in frozen brain slices of mice during sepsis. Human brain microvascular endothelial cells (HBMECs) were used to research the molecular mechanism of brain cell damage induced by P2X7R. The results showed that P2X7R inhibitors dramatically improved the survival rate of mice, relieved the cognitive dysfunction caused by LPS stimulation, and significantly reduced the brain cell apoptosis caused by LPS. In addition, the inhibition of P2X7R can also reduce the production and accumulation of reactive oxygen species (ROS) in HBMECs in vitro and inhibit the apoptosis signaling pathway associated with mitochondrial serine protease Omi/HtrA2 in HBMECs in vitro. These results suggest that P2X7R has strong value as a potential target for the treatment of SAE.

## 1. Introduction

SAE is an inflammatory infection that causes systemic sepsis to spread to the brain, but there is no evidence of direct infection in the brain, and this is one reason for long-term and short-term cognitive impairment and high mortality in sepsis patients in the ICU [1, 2]. More and more evidences show that SAE is closely related to peroxidation stress injury of brain cells, mitochondrial dysfunction, and apoptosis. Some researchers have found that 70% of severe sepsis survivors are accompanied by cognitive impairment [3, 4], but the pathogenesis of SAE is still unclear.

As a member in the P2X family of ATP-sensitive purinergic receptors, P2X7R is unique in that it only functions in homomeric forms and is activated by high extracellular ATP levels [5]. Activation of P2X7R causes a large amount of Ca2+ and Na+ influx and K+ outflow, resulting in the imbalance of intracellular metabolism. P2X7R is primarily expressed on the surface of various brain cells, especially microglia and astrocytes [6, 7]. Many scholars regard P2X7R as a receptor closely related to inflammatory injury, because P2X7R located in various organs of the body will be greatly activated when severe systemic infection occurs and the activation of P2X7R is also a damaging process [8]. In this regard, whether the brain damage caused by the activation of P2X7R in the brain can be considered as one of the mechanisms of SAE, and the molecular mechanisms in brain cells caused by the activation remain to be explored.

The blood-brain barrier (BBB) is a gateway through which the brain and the outside world exchange substances, allowing small molecules of nutrients and oxygen to enter the brain, while blocking harmful macromolecules from entering the brain [9]. It is well known that the BBB is mainly composed of cerebral capillary endothelial cells, basal membrane and foot processes of glial cells, and tight junction proteins produced by endothelial cells play a crucial role in the cell-cell connection of BBB and maintaining the integrity of BBB [10]. When harmful substances, such as lipopolysaccharide (LPS), arrive at BBB with blood flow, they will first damage endothelial cells, thereby inhibiting the expression of tight junction protein and damaging BBB [11]. However, it remains to be explored whether LPS-induced brain endothelial cell damage is related to P2X7R activation and whether inhibition of P2X7R has a protective effect.

Under normal physiology, the body's physiological metabolism will produce an appropriate amount of ROS, acting as a signal to regulate the physiological and biochemical reactions inside and outside cells of the body [12]. However, when the body suffers pathological injuries, such as invasion of external bacteria and reperfusion injury caused by ischemia and hypoxia of essential organs, the damaged organs will produce and accumulate ROS in large quantities. The accumulation of ROS in cells will destroy important organelles such as endoplasmic reticulum and mitochondria, even directly damage the DNA of cells [13, 14]. At present, many studies have found that the continuous production of ROS is closely associated with neurodegenerative diseases such as Parkinson's disease and Alzheimer's disease [15], as well as cell necrosis and apoptosis related to inflammation [16]. Although recent studies have found that the activation of P2X7R can induce ROS production in cardiomyocytes [17], the effect of P2X7R on the production of ROS in brain cells after severe sepsis-induced SAE remains to be explored. In addition, numerous studies have found that plenty of intracellular ROS can cause intracellular apoptosis related to caspase, Bcl-2, and cytochrome C [18]. However, whether ROS production can cause apoptosis related to mitochondrial serine protease Omi/HtrA2 remains unexplored.

Both apoptosis and programmed cell death occur in all organs of the body and maintain a balance between the removal of damaged cells and the renewal of new cells [19]. However, when the body is subjected to severe damaging stimuli, such as the disturbance of the body's internal environment caused by inflammation or biochemical stimulation, the cells of the body will undergo apoptosis caused by aspartate-specific cysteine proteases known as caspases. Cell apoptosis is closely related to mitochondria besides some signaling pathways in the cytoplasm [20]. Apoptosis caused by mitochondrial pathway is mainly related to the mitochondrial serine protease Omi/HtrA2, which exists in the inner and outer membrane of mitochondria under physiological conditions and is mainly involved in clarifying misfolded proteins in mitochondria [21, 22]. However, when cells are injured by various pathological factors such as external inflammation, biochemical and physiological changes will occur in the cells, thereby transferring Omi/HtrA2 from the mitochondrial intermembrane space into the cytoplasm [23, 24]. Nonetheless, it is still unclear whether the activation of P2X7R on the cell surface will affect the membrane potential of mitochondria and whether it will cause the transfer of Omi/HtrA2 from mitochondrial to cytoplasmic.

Apoptosis-related proteins XIAP, Caspase9, and Caspase3 play a vital role in the process of cell apoptosis [25]. As a member in the evolutionarily conserved family of inhibitors of apoptosis proteins (IAPs), X-linked inhibitor of apoptosis protein (XIAP) can bind and inhibit caspase-related apoptosis in cells [26]. When Omi/HtrA2 enters the cytoplasm from mitochondria, XIAP will be degraded, thus, inhibiting the degradation of caspase-9 by XIAP [27–29], and increased expression of caspase-9 activates caspase-3, ultimately causing intracellular DNA damage. It has been proved that cell apoptosis induced by external damaging stimuli is significantly reduced after mitochondrial serine protease Omi/HtrA2 is knocked down [30]. Therefore, the effect of P2X7R activation on XIAP and caspase-3-related apoptosis needs further exploration.

## 2. Materials and Methods

### 2.1. Reagents

Chemicals: P2X7R antagonist A-438079 and LPS were purchased from Sigma-Aldrich. Antibodies: Rabbit anti-cleaved-caspase-3, rabbit anti-cleaved-caspase-9, mouse anti-XIAP, mouse anti-P2X7R, Rabbit anti-ZO-1, Rabbit anti-Occludin, and mouse anti-HtrA2 were from Santa Cruz Biotechnology (Santa Cruz, CA, USA).

### 2.2. Animal Feeding and Animal Experiment

After obtaining the male C57BL/6 (8-10 weeks) mice from Medical Laboratory Animal Center of Weifang Medical University, they were allowed to adapt for seven days. The mice lived in a quiet room, with the rotation of dark and light every 12 hours, the room temperature of 25°C, and free access to food and water every day in the cage. All animal operations were conducted following ethical regulations and laboratory guidelines. To test whether A-438079 could affect LPS-induced SAE and cognitive function in mice, LPS was dissolved in PBS, and A-438079 was dissolved with 25% dimethyl sulfoxide (DMSO) (Sigma-Aldrich, USA). Then, the mice were divided into four groups. Control group mice were intraperitoneally injected with PBS of the same volume as LPS group. LPS group was injected intraperitoneally LPS (5 mg/kg), LPS + A − 438079 group was intraperitoneally injected with A-438079 (30 mg/kg) after one hour of pretreatment with LPS (5 mg/kg), LPS + DMSO group was intraperitoneally injected with DMSO after one hour of pretreatment with LPS (5 mg/kg). Body weight and the survival rate of the mice were recorded daily for 7 days (each group contained five mice), after that, the mice were decapitated and their brains were taken under pentobarbital anesthesia, and proteins were extracted from their hippocampal tissues for subsequent experiments. However, for behavioral tests, three days after the injection, behavioral tests were performed on each group of mice to determine the success of the model and the changes in cognitive function (each group contained ten mice). After the behavioral test (day 7 after LPS injection), the mice were placed under pentobarbital anesthesia with a heart infused with normal saline followed by 4% paraformaldehyde fixation and decapitated. Mouse brain tissue was immobilized in 4% paraformaldehyde for 4 h and then placed in 15% sucrose solution until the brain tissue sank to the bottom. The brain tissue was taken out, and frozen sections were performed after OTC embedding. The brain slices were stored at -20°C.

### 2.3. Cell Culture and Experiment

HBMEC (HUM-CELL-0101) cells were purchased from PriCells (Wuhan, China). The cells were cultured in DMEM medium of 30 mg/ml endothelial growth factor, 10% fetal bovine serum, and 10 mg/ml penicillin/streptomycin. Cells were cultured at 5% CO2 and 37°C. The culture medium was changed every 24 hours, and it was passed after 48 hours. Before LPS treatment, the cells were inoculated in a six-well plate at a density of 1 × 10^5^ cells/well and placed in an incubator overnight. On the second day, cells were pretreated with A-438079 (2.5 *μ*g/ml) or DMSO, and LPS (5 *μ*g/ml) was added one hour later in each group. After 12 hours of culture, the cells were collected for Western Blot analysis.

### 2.4. ROS Assay

The production of ROS in HBMEC was analyzed by DCFH-DA staining. According to the protocol of the manufacturer, DCFH–DA and DMEM are configured in quantities of 1 : 1000. The cells were inoculated in a six-well plate at a density of 1 × 10^5^ cells/well, and drugs are treated in line with the above cell treatment protocol, then, each well was added with 1 ml and incubated at 37°C for 40 minutes. Finally, ROS production in each group of cells was filmed by fluorescence microscopy (Olympus, Japan).

### 2.5. Mitochondrial Membrane Potential Analysis

We used Mito-Tracker Red CMXRos aggregation in mitochondria which depends on the characteristics of mitochondrial membrane potential to analyze the changes of mitochondrial membrane potential in different groups of cells. Briefly, HBMECs were inoculated in a six-well plate with preplaced circular slides overnight, followed by abandoning culture and adding 1 : 1000 Mito-Tracker Red CMXROS (purchased from Beyotime) working fluid to the preprepared medium at 37°C for 15-30 minutes, and finally, DAPI was added, with each group of cells filmed by fluorescence microscopy (Olympus, Japan).

### 2.6. Western Blot Analyses

Western Blot was roughly divided into four steps. The first step was electrophoresis. After adding the sample of each group to the electrophoresis tank, the electrophoretic liquid was added, and the electrophoresis is performed at 80 V for 30 minutes first, then, at 120 V for 60 minutes. In the second step of membrane transfer, the separation gel after electrophoresis was placed in a membrane transfer clamp with sponge and filter paper to transfer the membrane for 90 minutes in a transfer tank with the transfer solution at 300 mA. The third step was seal and incubation. After membrane transfer, the nitocellulose membrane was transferred to 5% skim milk powder for sealing for 2 hours, then, incubated overnight in the primary antibody at 4°C and in the secondary antibody at room temperature for 1-1.5 hours the next day. Fourth, ECL solution (purchased from Beyond) was added to the immunoreactive membrane and developed using Enhanced Chemiluminescent Detection. Results analysis: ImageJ software was used to analyze the gray value of the strip.

### 2.7. TUNEL Assay

The TUNEL fluorescence assay kit was used to detect apoptosis in frozen brain sections, i.e., a well-preserved frozen brain section was placed in a citrate solution in a microwave oven on high heat for 5 minutes, followed by medium heat for 10 minutes for percolation. Then, the section was washed three times with PBS solution and incubated for 60 min at 37°C under darkroom conditions after adding preconfigured TUNEL working solution (I solution : II solution = 1 : 9), followed by being washed three times with PBS and added DAPI to seal. TUNEL-positive cells were counted using image analysis software (Image-Pro Plus 6.0) under identical conditions, and each group of cells was recorded by fluorescence microscopy (Olympus, Japan).

### 2.8. Morris Water Maze

An operator who was not aware of each treatment group detected the cognitive function of mice by Morris water maze (MWM). The MWM consisted of a circular steel pool (125 cm in mice by Morris water maze (MWM)). The MWM consisted of a circular steel pool (125 cm in diameter, 60 cm in depth) with water depth below 1 cm above the top of the platform (10 cm in diameter, 30 cm in height). The pool was surrounded by a gray curtain and placed in a quiet room at 25°C with titanium dioxide to make the water turbid. MWM testing began on the third day after LPS or A-438079 treatment and lasted for five days, with the first 4 days (3-6 days) being a training period. The mice were released from various locations and given 60 seconds to find the platform, and they were artificially led to the platform and stayed there for 15 seconds if they failed to find the platform. A video surveillance system was used to record and track the escape latency of mice (i.e., the time taken from being placed in the water to finding the platform). On the 5th day (day 7), the mice were subjected to the space exploration experiment. The platform was removed, and the mice were released from the opposite side of the platform and swam free for 120 seconds, with the number of target crossings recorded.

### 2.9. Statistical Analyses

All data are presented in the mean-standard deviation, with their validation analyses were performed by SPSS24.0 (SPSS Inc., Chicago, IL). *P* < 0.05 was considered statistically significant. Differences among the groups were examined by one-way analysis of variance.

## 3. Results

### 3.1. P2X7R Inhibitors Can Increase the Survival Rate and Improve Cognitive Dysfunction in Mice with SAE

At present, little is known about the pathogenesis of SAE. Many studies have found that cells in the brain undergo apoptosis under the stimulation of inflammatory factors, which is crucial for the pathogenesis of SAE. Since P2X7R is a receptor closely associated with inflammation and amplifies inflammatory damage, we investigated the influence of P2X7R inhibitor A-438079 on cognitive function in mice after severe sepsis was induced by LPS.

**Figure 1:**
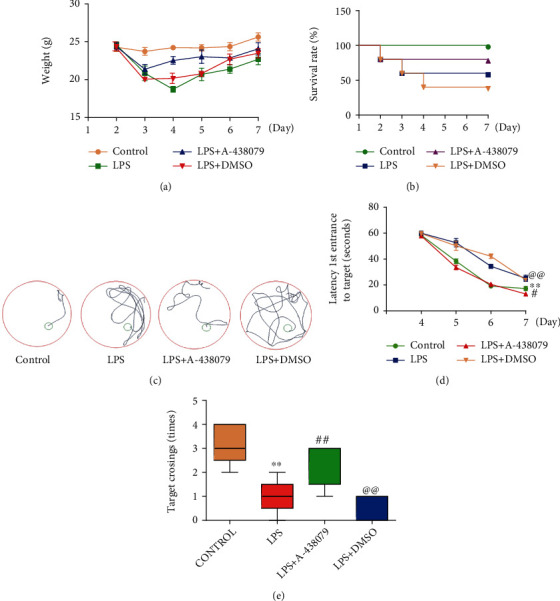
Treatment with P2X7R inhibitor A-438079 improved LPS-induced cognitive dysfunction and SAE in mice. (a) A-438079 treatment rescued LPS-induced weight loss. (b) Treatment of A-438079 significantly alleviated the reduced survival rate in mice caused by LPS. (c) The distance traveled by each group to reach the platform during the training period. Mice traveling distance was significantly reduced after treatment with A-438079 compared with the LPS and LPS+ DMSO groups. (d) Mice in LPS groups and LPS + DMSO groups expressed a signally longer escape latency than the control group. Treatment with A-438079 significantly reduced the extension of the escape latency after LPS treatment, *n* = 5. All data are expressed as mean ± SD. ^∗∗^*P* < 0.01 vs. control, ^#^*P* < 0.05 vs. LPS group, and ^@@^*P* < 0.01 vs. LPS + A − 438079 group. (e) The number of times the mice crossed the platform was significantly reduced in the LPS group compared with the control group but increased after treatment with A-438079 compared with the LPS group, moreover, the number of mice crossing the platform in the LPS + DMSO group was significantly decreased compared with the LPS + A − 438079 group; *n* = 5. All data are expressed as mean ± SD. ^∗∗^*P* < 0.01 vs. control, ^##^*P* < 0.01 vs. LPS group, and ^@@^*P* < 0.01 vs. LPS + A − 438079 group.

The results showed a significant weight loss after treatment with LPS compared to the control group, whereas the pretreated LPS group treated with A-438079 did not indicate a significant weight loss (Figure 1(a)). Similarly, we found a significant decrease in survival in mice after several weeks of LPS treatment compared to the control group, but pretreatment with A-438079 could reverse the LPS-induced low survival in mice (Figure 1(b)). When mice were trained for MWM (3-6 days), we found that the distance to the platform was significantly longer after LPS or LPS + DMSO treatment compared to the control group, while the distance was dramatically shortened after A-438079 treatment compared with those in LPS and LPS + DMSO groups (Figure 1(c)). Moreover, mice in the LPS treatment group and the LPS + DMSO group took notably longer to reach the platform during the training period than those in the control group, and the result was reversed after A-438079 treatment (Figure 1(d)). Then, the spatial memory test was performed on day 7 by removing the platform, releasing the mice from the quadrant facing the platform, and recording the number of times the mice crossed the platform area within 120 seconds. We found that the number of platform crossings was significantly reduced in the LPS group and the LPS + DMSO group compared with the control group, while the number in the A-438079 group was higher than that in the LPS and LPS + DMSO groups (Figure 1(e)).

From the above findings, we can find that LPS injection does cause cognitive impairment and lead to SAE in mice, while treatment with P2X7R inhibitor can improve the loss of spatial memory and directional learning abilities in mice. These results suggest that P2X7R plays a crucial role in the pathogenesis of SAE.

### 3.2. P2X7R Inhibitors Ameliorate LPS-Induced Tight Junction Protein Reduction in the Mouse Hippocampus

**Figure 2:**
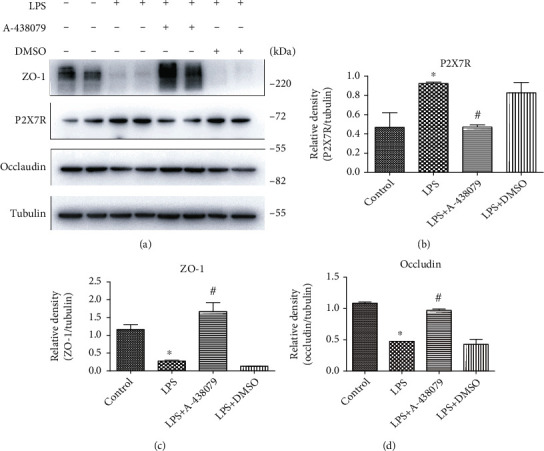
Treatment with the P2X7R inhibitor A-438079 improved LPS-induced decline in tight junction protein expression in the mouse hippocampus. (a) The expression levels of ZO-1, P2X7R, and Occludin were detected by Western Blot. Respective bands with Tubulin as loading controls are shown. (b) The expression of P2X7R was significantly increased in the hippocampus of mice after intraperitoneal injection of LPS. All data are expressed as mean ± SD. ^∗^*P* < 0.05 vs. control. (c) and (d) LPS significantly inhibited the expression of tight junction proteins ZO-1 and Occludin in the hippocampal tissues of mice, while A-438079 reversed this effect. All data are expressed as mean ± SD. ^∗^*P* < 0.05 vs. control, and ^#^*P* < 0.05 vs. LPS group.

In order to verify the protective effect of P2X7R inhibitor on BBB in the hippocampal area of mice after LPS treatment, we extracted the protein from the hippocampal tissue of mice and detected the expression of tight junction protein in each group by Western Blot. The results showed that LPS treatment significantly increased the expression of P2X7R in the hippocampus of mice (Figures 2(a) and 2(b)), and compared with the control group, LPS treatment significantly reduced the expression of tight junction protein Occludin and ZO-1, while LPS + A − 438079 group reversed this effect (Figures 2(a), 2(c), and 2(d)). From these results, it is clear that P2X7R plays a key role in LPS-induced BBB damage in mice.

### 3.3. P2X7R Inhibitors Can Attenuate the ROS Production and Changes in Mitochondrial Membrane Potential in HBMECs Induced by LPS Stimulation In Vitro

**Figure 3:**
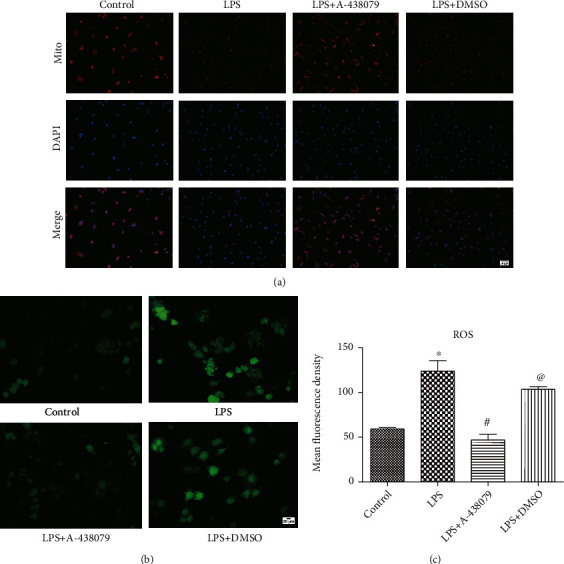
Effects of LPS or A-438079 on ROS production and mitochondrial membrane potential in HBMECs in vitro. (a) The adherent HBMECs in the LPS + A − 438079 group were treated with A-438079 for an hour and then treated with LPS for 12 hours. The same cells in the LPS + DMSO group were treated with DMSO for an hour and then treated with LPS for 12 hours. The LPS group was treated with LPS one hour after the addition of the same amount of normal saline for 12 hours. Mito-tracker Red CMXROS was used to evaluate the mitochondrial membrane potential in each group, and the intensity of red fluorescence signal in each group indicated the change of mitochondrial membrane potential. Scale bars = 50 *μ*m. (b) A fluorescence microscope was used to observe the production of ROS in each group. Scale bars = 20 *μ*m. (c) The fluorescence signals of different groups were analyzed using ImageJ. All data are expressed as mean ± SD. ^∗^*P* < 0.05 vs. control group, ^#^*P* < 0.05 vs. LPS group, and ^@^*P* < 0.05 vs. LPS + A − 438079 group.

As the activation of P2X7R will lead to a large increase in intracellular Ca^2+^ level, HBMEC cells cultured in vitro were selected and given LPS or P2X7R inhibitor A-438079 to research the effect of P2X7R activation on intracellular mitochondrial membrane potential and ROS production. Then, the production of ROS in HBMECs was analyzed by DCFH-DA staining, and the fluorescence intensity of each group was analyzed by ImageJ. The results showed that HBMECs produced more ROS when stimulated by LPS than by normal saline alone, and this phenomenon was reversed by P2X7R inhibitor A-438079 (Figures 3(b) and 3(c)). Since A-438079 was dissolved in DMSO, we found that the LPS + DMSO group was not statistically significant compared with the LPS group.

Since the fluorescence emitted by Mito-tracker Red CMXROS is dependent on mitochondrial membrane potential, we then used Mito-tracker Red CMXROS in HBMECs in vitro to evaluate the intracellular mitochondrial membrane potential. We found that the fluorescence signal intensity of the LPS stimulation group alone or the LPS + DMSO group was observably decreased than that of the control group and the LPS + A − 438079 group (Figure 3(a)). It can be seen that when brain cells are stimulated by inflammation, P2X7R inhibitors can reduce intracellular ROS production and have a protective effect on mitochondria.

### 3.4. LPS Stimulation Induced the Transfer of Omi/HtrA2 from Mitochondria to the Cytoplasm In Vitro and P2X7R Inhibitors Inhibited This Process

**Figure 4:**
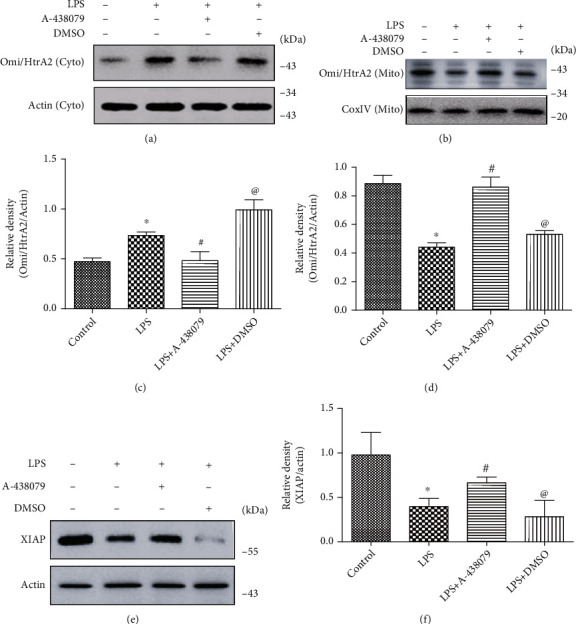
A-438079 treatment decreased LPS-induced transposition of Omi/HtrA2 from mitochondria to cytoplasm and reversed the decrease in XIAP expression. (a) and (c) A-438079 reduced LPS-induced increase of Omi/HtrA2 in the cytoplasm of HBMECs in vitro. All data are expressed as mean ± SD. ^∗^*P* < 0.05 compared with control, ^#^*P* < 0.05 compared with LPS, and ^@^*P* < 0.05 compared with LPS + A − 438079. (b) and (d) Under the effect of LPS, the mitochondrial Omi/HtrA2 of HBMECs in vitro was significantly decreased compared with the control group, and this reduction could be inhibited by A-438079. All data are expressed as mean ± SD. ^∗^*P* < 0.05 compared with control, ^#^*P* < 0.05 compared with LPS, and ^@^*P* < 0.05 compared with LPS + A − 438079. (e) and (f) Western Blot showed that LPS treatment could inhibit the expression of the apoptosis inhibitor protein XIAP and could be reversed by A-438079. All data are expressed as mean ± SD. ^∗^*P* < 0.05 compared with control, ^#^*P* < 0.05 compared with LPS, and ^@^*P* < 0.05 compared with LPS + A − 438079.

We speculated that the large influx of calcium ions caused by P2X7R activation would lead to the apoptosis of mitochondrial pathways, i.e., the Omi/HtrA2 protease generally presented in the mitochondria probably be transferred into the cytoplasm under the inflammation stimuli. Therefore, we explored the influence of P2X7R on the intracellular localization of Omi/HtrA2 cells under inflammatory conditions. We extracted the proteins in the cytoplasm and mitochondria of HBMECs cells by the mitochondrial extraction kit and found that under the action of LPS, there was the evident translocation of Omi/HtrA2 into the cytoplasm from mitochondria in HBMECs. However, when P2X7R inhibitor A-438079 was given in advance and then treated with LPS, reversal of Omi/HtrA2 translocation was observed (Figures 4(a) and 4(c)). Nevertheless, the LPS-induced transfer of Omi/HtrA2 to the cytoplasm was reversed after the treatment of A-438079. (Figures 4(a)–4(d)).

These results indicate that P2X7R expression in cells is significantly increased under inflammatory stimulation, and P2X7R inhibitors can alleviate the cytoplasmic translocation of apoptosis-related protein Omi/HtrA2 in mitochondria induced by LPS. Therefore, we further researched the effect of A-438079 on the downstream signaling pathway of Omi/HtrA2-induced apoptosis.

Previous studies have shown that XIAP can inhibit apoptosis by degrading apoptotic protein caspase-3 and caspase-9, while Omi/HtrA2 protease mainly degrades XIAP and thus leading to increased activation of caspase-3 and caspase-9. Therefore, in sepsis-associated encephalopathy caused by LPS, P2X7R activation may induce the brain cell apoptosis through the Omi/HtrA2 protease signaling pathway.

In order to identify the impact of P2X7R inhibitor on apoptosis-related proteins, the expression of apoptosis inhibiting protein XIAP was detected by Western Blot. As previously predicted, the level of XIAP in HBMECs was significantly higher in the inhibitor A-438079 group than in the LPS (Figures 4(e) and 4(f)).

### 3.5. P2X7R Inhibitors A-438079 Alleviated Apoptosis of Brain Cells after LPS Stimulation In Vivo and Reduced Cleaved Caspase-3 and Cleaved Caspase-9 Expression

Caspases are a class of cysteine proteases that exist in the cytoplasm and play an important role in the upstream and downstream of cell death signaling pathways. Caspase-9, for example, is activated by self-shearing in response to a stimulus signal, which in turn activates downstream apoptotic agents such as Caspase-3.

**Figure 5:**
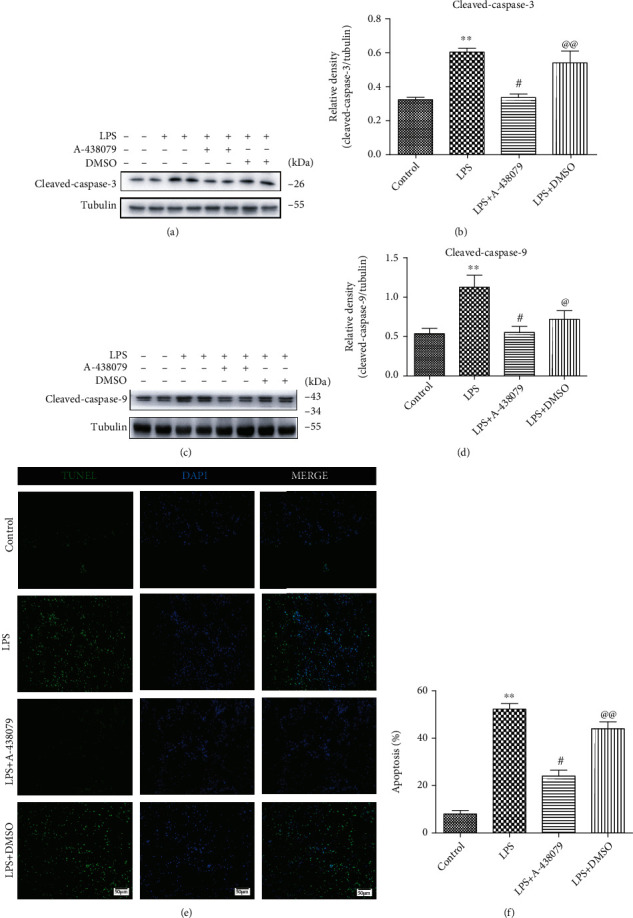
A-438079 inhibited Cleaved caspase-9 and Cleaved caspase-3 expression upregulation induced by LPS stimulation and LPS-induced apoptosis of brain cells in mice. (a)–(d) LPS-induced the increasing of Cleaved caspase-9 and Cleaved caspase-3 expression was inhibited by A-438079. All data are expressed as mean ± SD. ^∗∗^*P* < 0.01 compared with control, ^#^*P* < 0.05 compared with LPS, ^@@^*P* < 0.01 compared with LPS + A − 438079. (e) and (f) TUNEL fluorescence staining shows apoptosis of cells in the brain of mice. Scale bars = 50 *μ*m. All data are expressed as mean ± SD. ^∗∗^*P* < 0.01 compared with control, ^#^*P* < 0.05 compared with LPS, and ^@@^*P* < 0.01 compared with LPS + A − 438079.

In order to explore the molecular mechanism of P2X7R induced apoptosis of brain cells by LPS stimulation, we first pretreated microglia with P2X7R inhibitor A-438079 in vitro, followed by LPS stimulation. The expression of cleaved-Caspase-9 and cleaved-Caspase-3 in the LPS + A − 438079 groups was decreased compared with the LPS and LPS + DMSO groups (Figures 5(a)–5(d)), while these two indicators were not statistically significant in the inhibitor A-438079 group compared with the control group (Figures 5(a)–5(d)). In order to observe the effect of LPS and A-438079 on the brain cell apoptosis in mice, we further selected frozen brain sections of mice for TUNEL staining. The results demonstrated that a large number of brain cell apoptosis occurred in mice after the administration of LPS 5 mg/kg compared with the control group. However, the number of apoptotic cells in the brain of mice treated with A-438079 after LPS injection was significantly reduced comparing with the LPS group and the LPS + DMSO group. (Figures 5(e) and 5(f)). These results suggest that P2X7R inhibitor A-438079 can reduce intracellular Cleaved Caspase-9 and Cleaved Caspase-3 protein content and also curb the brain cell apoptosis during sepsis in mice.

## 4. Discussion

Being a syndrome involved multiple brain disorders, SAE is defined by many studies as cognitive impairment caused by severe peripheral sepsis or direct damage to brain tissue without evidence of direct infection in the brain (e.g., hepatic or renal encephalopathy) [31]. Cerebral dysfunction caused by septicemia has been a vital cause of delirium or altered mental state in critically ill patients [32]. Moreover, SAE can dramatically increase the mortality rate of sepsis patients [33, 34]. The destruction of the blood-brain barrier is crucial in SAE, and it leads to the activation of a series of cell surface receptors (especially the P2X7R) in the brain, resulting in the damage of reactive oxygen species and the occurrence of apoptosis and necrosis in the brain [35].

Among the components of BBB, tight junction protein plays an important role in promoting the tight binding between cells and the transmission of information between cells. The relatively common tight junction proteins mainly include transmembrane proteins, such as Occludin, Claudin, and JAM-1, and cytoplasmic attachment proteins, such as ZO-1 [36]. Our study found that LPS-induced inflammatory stimulation activated P2X7R in the hippocampus of mice and further reduced tight junction protein expression, whereas when we used P2X7R inhibitor A-438079, the expression of ZO-1 and Occludin was significantly improved. Therefore, inhibition of P2X7R can protect the integrity of the blood-brain barrier to some extent and reduce the damage of brain cells.

Previous studies have found that the mitochondrial serine protease Omi/HtrA2 plays a critical role in the pathogenesis of SAE [29, 35], but their study was limited to Omi/HtrA2 itself. In this study, we found that P2X7R is crucial for cytoplasmic translocation of Omi/HtrA2 under inflammatory stimulation, and there are two possible reasons. First, our experiment found that after activation of P2X7R, plenty of ROS would be produced in cells, causing severe damage to mitochondria in cells. This damage causes the opening of mitochondrial outer membrane channels (especially mPTP) [37], allowing Omi/HtrA2 to enter the cytoplasm. However, as a critical ROS producer, mitochondria also produce bountiful ROS when stimulated, thus, amplifying this effect. Second, as a nonselective cation channel on the cell surface, P2X7R will cause numerous cation influx after activation, break the ion balance inside and outside the cell, and also destroy the membrane potential of mitochondria, leading to cell apoptosis.

In this study, we found that after intraperitoneal injection of LPS, mice had significant cognitive dysfunction in the short term, especially spatial memory and directional sense, and all of them could be alleviated by the treatment of P2X7R inhibitor A-438079. Studies have found that the activation of P2X7R in the brain under inflammatory stimulation can not only result in the release of inflammatory factors (such as IL-6 and TNF-*α*) [6], but also activate some signaling pathways (such as NLRP3/IL-1*β* and STAT3) [38, 39]. However, few studies have been conducted on the role of P2X7R in mediating apoptosis of brain cells under inflammatory conditions. Our study found a mitochondria-related apoptosis pathway caused by P2X7R activation, providing some clues to the possible pathogenesis of SAE.

**Figure 6:**
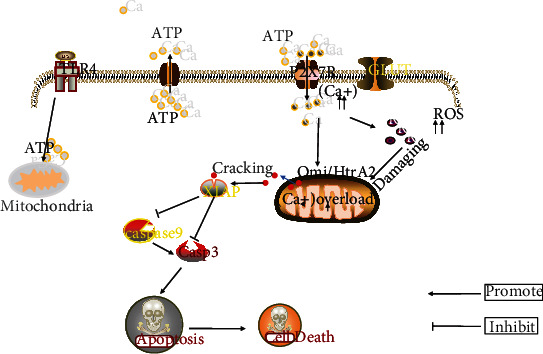
LPS stimulated cells to induce the P2X7R-mediated intracellular Omi/HtrA2 apoptosis signaling pathway. Under the stress stimulation of LPS, mitochondria in cells will produce a large amount of ATP and activate P2X7R on the cell surface. The activated P2X7R will cause a large amount of Ca2+ influx, and then the imbalance of intracellular Ca2+ homeostasis will lead to the production of a large amount of intracellular ROS and further cause mitochondrial damage. However, Ca2+ overload in mitochondria will further increase the production of ROS and cause the transfer of OMI/HtrA2 in the damaged mitochondrial membrane space into the cytoplasm. Omi/HtrA2 in the cytoplasm can crack the apoptosis inhibiting protein XIAP, which leads to the decrease of XIAP inhibiting caspase-3 and caspase-9 in cells, and promotes cell apoptosis.

To sum up, our data demonstrate that P2X7R plays a damaging role in the pathogenesis of SAE. Besides, we provided some valuable evidence, for example, P2X7R activates brain cells to produce a lot of ROS in vivo sepsis, mediates mitochondrial damage and Omi/HtrA2 translocation from mitochondria to cytoplasm, thereby promoting the cleavage of the intracellular apoptosis-inhibiting protein XIAP through Omi/HtrA2, and increases the expression of proapoptotic protein such as cleaved-Caspase-3 (Figure 6), ultimately leading to cognitive impairment. These findings provide potential therapeutic targets for the treatment of encephalopathy caused by severe infectious diseases and probably reduce the clinical mortality of SAE patients in the future.

## 5. Conclusion

In this study, we found that P2X7R inhibitors can protect the blood-brain barrier in mice and alleviate the ROS production in brain cells induced by LPS stimulation. In addition, A-438079 also inhibits cell apoptosis by blocking mitochondrial serine protease transfer from mitochondria into the cytoplasm. Moreover, we also demonstrated that P2X7R inhibitors can significantly treat short-term cognitive dysfunction caused by LPS in mice. These findings further demonstrate the great importance of P2X7R in the clinical treatment of SAE.

## Data Availability

The data used to support the findings of this study are available from the corresponding author upon request.
